# Supplementary material for: Characterization of a putative NsrR homologue in Streptomyces venezuelae reveals a new member of the Rrf2 superfamily
Source: Sci Rep. 2016 Sep 8;6:31597. doi: 10.1038/srep31597 (PMC5015018; doi:10.1038/srep31597)
Supplement: Supplementary Information [file srep31597-s1.doc]

**Characterization of a putative NsrR homologue in *Streptomyces venezuelae* reveals a new member of the Rrf2 superfamily**

John T. Munnoch^1^, M^a^ Teresa Pellicer Martinez^2^, Dimitri A. Svistunenko^3^, Jason C. Crack^2^, Nick E. Le Brun^2#^ and Matthew I. Hutchings^1#^

^1^School of Biological Sciences, University of East Anglia, Norwich, Norwich Research Park

^2^Centre for Molecular and Structural Biochemistry, School of Chemistry, University of East Anglia, Norwich, Norwich Research Park

^3^School of Biological Sciences, University of Essex, Wivenhoe Park, Colchester

**Correspondance:** [**m.hutchings@uea.ac.uk**](mailto:m.hutchings@uea.ac.uk)**,** [**n.le-brun@uea.ac.uk**](mailto:n.le-brun@uea.ac.uk)


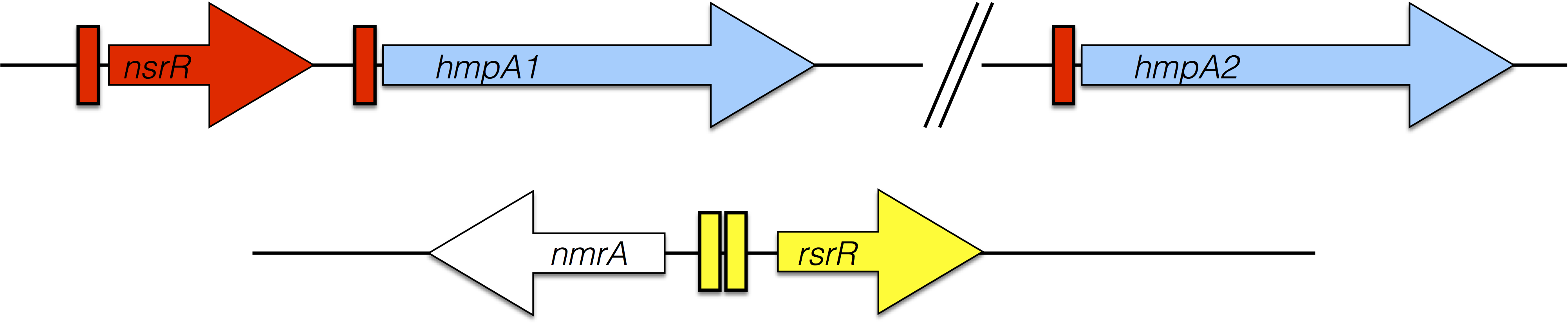


**Figure S1. *Streptomyces* NsrR proteins are genetically linked to genes encoding the NO dioxygenase** HmpA. Shown here (top) in *S. coelicolor* where ScNsrR (red) regulates itself and both HmpA (blue) homologues and is linked to *hmpA1*. ScNsrR binding sites are shown as red boxes. *S. venezuelae* does not encode an HmpA homologue and Sven6563 (RsrR, yellow) is divergently transcribed from *sven6562* (NmrA, white) which encodes a LysR family regulator with an NmrA-type NAD/NADP binding domain. NmrA (PMID: 12764138) is a transcriptional repressor in fungi which can distinguish between oxidised and reduced NAD and NADP and may be a redox sensor. Both *nmrA* and *rsrR* are repressed by RsrR binding (yellow boxes) at two 25 bp sites separated by 1 bp.

**Figure S2. Illustration of the *nmrA-rsrR* promoter region including the RsrR binding sites.** (a) A graphical representation of the 107 bp promoter region for *nmrA* (*sven6562*) and *rsrR* (*sven6563*) along with the sequence showing the two class 1 binding sites (purple (motifs 1 and 2) and orange (motifs 3 and 4)) and the flanking sequences (blue and green). (b) A graphical representation indicating the binding sites used for the Figure 5 promoter probes. Down the left (1) = Figure 5b 1, (2) – Figure 5b 2, (3) Figure 5c 1, (4) Figure 5c 2, (5) Figure 5c 3, (6) Figure 5c 4.


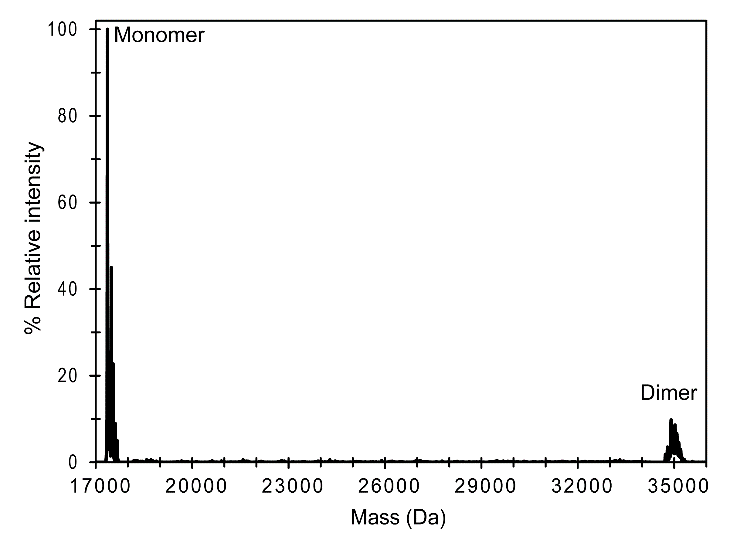


**Figure S3. Full range native mass spectrum of RsrR**. Positive ion mode ESI-TOF native mass spectrum of ~21 µM [2Fe-2S] RsrR in 250 mM ammonium acetate pH 8.0, The full *m*/*z* spectrum was deconvoluted with Bruker Compass Data analysis with the Maximum Entropy plugin.
